# Anti-Inflammatory and Anti-Fibrotic Profile of Fish Oil Emulsions Used in Parenteral Nutrition-Associated Liver Disease

**DOI:** 10.1371/journal.pone.0115404

**Published:** 2014-12-12

**Authors:** Alfonso Pastor-Clerigues, Ezequiel Marti-Bonmati, Javier Milara, Patricia Almudever, Julio Cortijo

**Affiliations:** 1 Hospital Pharmacy, Clinical Nutrition Unit, University General Hospital Consortium, Valencia, Spain; 2 Clinical research unit (UIC), University General Hospital Consortium, Valencia, Spain; 3 Research Foundation of General Hospital of Valencia, Valencia, Spain; 4 Department of Pharmacology, Faculty of Medicine, University of Valencia, Valencia, Spain; 5 CIBERES, Health Institute Carlos III, Valencia, Spain; National Institutes of Health, United States of America

## Abstract

Home parenteral nutrition (PN) is associated with many complications including severe hepatobiliary dysfunction. Commercial ω-6 fatty acid-soybean based-lipid emulsions in PN may mediate long term PN associate liver disease (PNALD) whereas ω-3-fish oil parenteral emulsions have shown to reverse PNALD in children. However, its clinical effectiveness in adults has been scarcely reported. In this work, we study the role of soybean and fish oil lipid commercial emulsions on inflammatory and profibrotic liver markers in adults with long term PNALD and in *in vitro* cellular models. Inflammatory and profibrotic markers were measured in serum of ten adults with long term PNALD and in culture supernatants of monocytes. Liver epithelial to mesenchymal transition (EMT) was induced by transforming growth factor beta 1 (TGFβ1) to evaluate *in vitro* liver fibrosis. Omegaven®, a 100% fish oil commercial emulsion, was infused during four months in two patients with severe long term PNALD reversing, at the first month, the inflammatory, profibrotic and clinical parameters of PNALD. The effect was maintained during the treatment course but impaired when conventional lipid emulsions were reintroduced. The other patients under chronic soybean oil-based PN showed elevated inflammatory and profibrotic parameters. *In vitro* human monocytes stimulated with lipopolysaccharide induced a strong inflammatory response that was suppressed by Omegaven®, but increased by soybean emulsions. In other experiments, TGFβ1 induced EMT that was suppressed by Omegaven® and enhanced by soybean oil lipid emulsions. Omegaven® improves clinical, anti-inflammatory and anti-fibrotic parameters in adults with long-term home PNALD.

## Introduction

The introduction of long-term parenteral nutrition (PN) has profoundly impacted the prognosis and quality of lives of patients unable to absorb adequate enteral nutrients, usually secondary to intestinal failure from congenital abnormalities or extensive gastrointestinal surgery [1]. Long-term use of PN, however, is associated with many complications including septic infection, metabolic imbalance and hepatobiliary dysfunction [2]. The hepatobiliary complications of PN are now well recognized as PN associated liver disease (PNALD). The clinical spectrum includes steatosis, cholestasis, cholelithiasis, hepatic fibrosis, and ultimately progression to biliary cirrhosis, portal hypertension, and end-stage liver failure [3]. Approximately 15% of adult patients who receive long-term PN eventually develop end-stage liver disease [4]. PN is typically infused as a mixture of micronutrients such as vitamins electrolytes and trace elements, and macronutrients such as amino acids, glucose and lipids that provide a source of non-protein calories and prevent essential fatty acid deficiency. Mounting evidence indicates that PNALD may in part be due to the components of the conventional soybean oil–based lipid emulsions that were introduced more than forty years ago. In this regard, it has been shown that long-term use of a soybean-based lipid emulsion leads to a progressive increase of cholestasis in children on long-term PN [5]. Furthermore ω-6 polyunsaturated fatty acids (PUFAs) soybean-based lipid emulsions are thought to be proinflammatory, oxidative and immunosupresive, and the content in phytosterols may also produce hepatotoxic effects, altogether contributing to PNALD [6]. In order to counteract deleterious effects of soybean-based lipid emulsions, recent parenteral lipid emulsions have included other oil sources such as coconut, olive and fish oils, mixed in different proportions with soybean oils. Thus, olive oil is rich in ω-9 monounsaturated fatty acids (MUFAs), which have been shown to preserve immune function. These formulations also contain the antioxidant α-tocopherol which could reduce inflammation and oxidative stress generated by ω-6 PUFAs present in the same formulation [7].

Fish oil–based lipid emulsions are the most recent development as an alternative to soybean oil. Due to its high concentration of eicosapentaenoic acid (EPA, ω-3) and docosahexaenoic acid (DHA, ω-3), fish oil has been known to have anti-inflammatory potential by interfering with the arachidonic acid pathway by inhibiting cytokines that trigger proinflammatory reactions [8]. In animal models of PNALD, parenteral fish oil administration improves biliary flow and cholestasis [9]. In humans, several nonrandomized human investigations have accumulated substantial data to support the beneficial effects of ω-3-rich fatty acids in the treatment of PNALD in infants [10], however there is a lack of information in adults developing PNALD secondary to long-term PN, where PNALD has a different etiology [11].

The plant-based lipid emulsions Intralipid® (100% soybean oil, Baxter/Fresenius Kabi), and Lipofundin MCT/LCT® (50% soybean oil, 50% coconut oil, B.Braun) were the first lipid emulsions available for PN, and currently the only approved (with Liposyn II®) for clinical use in United States. Recently, two new lipid emulsions were introduced. Olive-based ClinOleic® (20% soybean oil, 80% olive oil, Baxter) and fish oil SMOFlipid® (30% soybean oil, 30% coconut, 25% olive oil, and 15% fish oil, Fresenius Kabi) fully cover the recommended essential fatty acid requirements, and provides a superior safety profile than the observed for the older lipid formulations.

Omegaven® (Fresenius Kabi) was introduced as a 100% fish oil lipid emulsion that, although does not provide the required essential fatty acid load, it has been used in short period of times as monotherapy or in addition to other lipids of PN to treat liver complications. However, Omegaven® is used mainly in compassionate-use protocols in the treatment of PNALD because the lack of controlled clinical trials.

In the present study we describe the effect of different soybean and fish parenteral lipid emulsions in adults who developed PNALD secondary to long-term PN. In addition we analyze their anti-inflammatory effects on human monocytes and their anti-fibrotic effects on human hepatocytes. Results provided in this work may be of potential value to appropriately select the optimal parenteral lipid treatment to minimize PNALD and its end-stage liver complications.

## Materials and Methods

### Ethics Statement

The present study was conducted according to the guidelines laid down in the Declaration of Helsinki, and all procedures involving human subjects were approved by the General University Hospital of Valencia, Spain ethics committee (CEIC reference no. R19/12/2013). An informed written consent was received from all patients and healthy subjects.

### Patients and Study Design

This is a proof of concept observational follow up clinical study carried out on adult patients with long-term home PN due to a short bowel syndrome after massive surgical resection due to non-oncologic/inflammatory complications. Since this is a proof of concept study based in clinical practice, the sample size was not calculated and patients studied were only from the General University Hospital, Valencia Spain.

All patients studied showed the following characteristics: 1) age>18 years old; 2) Presence of non-alcoholic steatohepatitis (NASH), classified according to the non-alcoholic fatty liver disease (NAFLD) activity score (NAS) system previously outlined [12]; 3) Diagnosis of PNALD once discarded full viral hepatitis serologies, autoimmune serologies, iron studies, α-1 antitrypsin level, and ceruloplasmin positivities and other causes different to PN; 4) ≥ of 2 years of home PN; 5) Patients with parenteral lipid emulsion Lipofundin MCT/LCT®, ClinOleic® or SMOFlipid®. None of the patients showed: 1) age <18 years old; 2) absence of NASH; 3) Other concomitant cause of liver disease different to PNALD; 4) ≤ of 2 years of PN.

All patients under long-term home PN at the clinical nutrition unit were revised for clinical evolution following internal standard-of-care guidelines in which patients with NAS score>6 due to PNALD were selected to receive 100% fish-oil Omegaven® 1 g/kg (5 PN/week) as lipid monotherapy in their PN during 4 months, followed by a change to SMOFlipid® 1 g/kg (4 PN/week) parenteral lipid emulsion for additional 2 months according to clinical practice. For comparison, healthy subjects and home PN patients with NAS score ≤6 under chronic Lipofundin MCT/LCT® (1 g/kg; 4 PN/week), ClinOleic® (1.2 g/kg; 4 PN/week) or SMOFlipid® 1 g/kg (4 PN/week) parenteral lipid emulsions were recruited to measure basal clinical and laboratory parameters. Supporting STROBE checklist is available as supporting information (Checklist S1).

### Classification of Liver Biopsy and Laboratory Determinations

Liver biopsies were sampled to determine NAS and hepatic fibrosis scores. Samples were graded according to the NAS system proposed previously [12]. Briefly, grade of steatosis was scored as S0≤5%; S1: 5%–33%; S2≥33–66%: S3>66%; grade of lobular inflammation was scored as 0 = no foci; 1≤2 foci/200xfield; 2 = 2-4 foci/200xfield; 3>4 foci/200xfield; and grade of ballooning was scored as 0 =  none; 1 =  few ballooning cells; 2 =  many cells/prominent ballooning. The grade of steatosis (0-3), lobular inflammation (0-3), and ballooning (0-2) were then combined to determine the NAS activity score (0-8). Fibrosis was scored as F0  =  none; F1  =  periportal or perisinusoidal fibrosis; F2  =  perisinusoidal and portal/periportal fibrosis; F3  =  bridging fibrosis; and F4  =  cirrhosis. Blood samples were analysed for aspartate transaminase (AST), alanine aminotransferase (ALT), alkaline phosphatase (ALP); gamma glutamyl transferase (GGT), bilirubin, albumin, prealbumin, total protein, platelets, hemoglobin, leucocytes, neutrophils, monocytes and lymphocytes by routine hospital methods.

### Determination of Fibrotic and Inflammatory Markers in Serum and Cell Culture Supernatants

Inflammatory and profibrotic markers such as matrix metalloproteinase 9 (MMP9), tumor necrosis factor alpha (TNFα), interleukin 1 beta (IL-1β), IL-2, IL-4, IL-5, IL-6, IL-8, IL-10, IL-12 and granulocyte-macrophage colony-stimulating factor (GM-CSF) were measured in serum and in cell culture supernatants by LUMINEX technology according to the manufacturer's protocol.

Quantitative ELISA for TGF-β1 was performed in serum as well as with supernatants of human monocytes stimulated with LPS during 24 h in presence or absence of parenteral lipid emulsions using a quantikine human TGF-β1 immunoassay (R&D Systems). To measure latent complexes of TGF-β1, activation was accomplished by acid treatment. Therefore, 50 µl of serum or cell culture supernatants were treated with 10 µl of 1 mol/l HCl, incubated for 10 min, and then neutralised with 10 µl of 1.2 mol/l NaOH/0.5 mol/l HEPES.

### Monocyte Isolation and Stimulation

Monocytes were isolated from peripheral venous blood (∼40 mL) of healthy subjects. Peripheral venous blood was mixed with dextran 500 at 3% (in 0.9% saline) in a proportion of 2∶1. This mixture was incubated at room temperature for 30 min until erythrocytes were sedimented. The upper phase was carefully collected and added on Ficoll-Paque Histopaque 1077 (Amershan Pharmacia Biotech, Barcelona, Spain) density gradient in a proportion of 3∶1. The two phases generated were centrifuged at 150 g, 4°C for 30 min. Thus, the interface was collected to isolate mononuclear cells. Cell suspension was washed two times with phosphate buffer (PBS). The interface cell suspension was adjusted to 500×10^3^ cells per well in 24-well plates and incubated for 4 h before non-adherent cells were discarded and remaining cells were kept in RPMI 1640 culture medium containing 0.25% FCS for at least 6 h before stimulation.

The preparations were>96% in monocytes, as assessed by Giemsa staining, and had a viability of>99%, measured by trypan blue exclusion. Neither purity nor viability was affected in the study's different experimental conditions.

Human monocytes were adjusted to 500×10^3^ cells per well in 24-well plates and treated in presence or absence of Omegaven® 10% (Fresenius Kabi, Steinbach, Germany), ClinOleic® 20% (Clintec parenteral S.A, Maurepas, France), Lipofundin MCT/LCT® 20% (B. Braun Medical S.A, Barcelona, Spain) or SMOFlipid® 20% (Fresenius Kabi, Barcelona, Spain) at dilutions of 1/10, 1/100 and 1/1000 in RPMI 1640 culture medium for 30 min before the stimulation with lipopolysaccharide (LPS; 1 µg/ml). Final dilutions of parenteral lipid emulsions for *in vitro* studies were selected based on pharmacokinetic values described in lipid emulsion technical manuals provided by manufacturers. A dilution of 1/100 results in a fatty acid concentration of 200 mg/dL which is close to the plasma concentrations described following 1.2 g/kg parenteral lipid infusion in humans [13]. We selected LPS 1 µg/mL as endotoxin stimulus related with monocyte activation and liver fibrosis [14]. LPS concentration was selected because is widely used in the literature for this type of studies [15], [16], [17], and because we obtained a ∼80% of maximal response of IL-8 and MMP-9 release from monocytes at LPS 1 µg/mL concentration in preliminary studies.

Both the stimuli and drugs remained together for 24 h. Supernatants were collected and centrifuged at 120 g for 5 min, and the cell-free supernatant was used to measure different proinflammatory and profibrotic markers described in this section.

### Liver Epithelial Cell Culture and Stimulation

The human liver epithelial cell line, THLE-3, was purchased from the American Type Culture Collection (Manassas, Va). The cells were maintained in collagen-coated culture dishes (10 µg cm^−2^ rat type I collagen (Sigma, Madrid, Spain) in epithelial growth medium (BEGM; Lonza, Madrid, Spain) as recommended by manufacturer, and incubated at 37°C and 5% CO_2_. Medium was changed every 2 to 3 days. Experiments on liver epithelial cells were selected to study the effects of different parenteral lipid emulsions on the epithelial to mesenchymal transition (EMT) process as a liver fibrotic mechanism previously described [18]. The profibrotic mediator transforming growth factor beta 1 (TGFβ1) was selected as a potent and universal inductor of fibrosis and EMT. In this regard, cells undergoing EMT, loss the epithelial phenotype and gain mesenchymal/myofibroblast phenotype, which includes an elevated migratory capacity and secretion of extracellular matrix components such as collagen type I and IV contributing to liver fibrosis [18]. In this study, THLE-3 cells were treated with Omegavent® 10%, ClinOleic® 20%, Lipofundin MCT/LCT® 20% or SMOFlipid® 20% at final dilutions of 1/10, 1/100 and 1/1000 in BEGM culture medium for 30 min before the stimulation with TGFβ1 5 ng/mL. Stimulus and lipid emulsions remained together for 72 h. At this time, total RNA and protein were extracted to study EMT and the mechanisms implicated. Immunofluorescence and visible microscopy was performed to analyze alpha smooth muscle (αSMA) expression and distribution, collagen type I (Col type I) expression and cell morphology.

### Histological and Immunofluorescence

Human liver biopsies were collected for diagnosis purposes according clinic normal procedures. Liver biopsies were fixed in paraformaldehyde 4% and included in parafine blocks. Tissue blocks (4 µm thickness) were stained with haematoxylin-eosin for assessment of liver tissue morphology and with masson's trichrome (Sigma-Aldrich, Madrid, Spain) to detect collagen deposition.

Human liver epithelial THLE-3 cell line were washed three times with PBS and fixed (4% paraformaldehyde, 30 min, at room temperature). After another three washes with PBS, THLE-3 cells were permeabilized (20 mM HEPES pH 7.6, 300 mM sucrose, 50 mM NaCl, 3 mM MgCl2, 0.5% Triton X-100), blocked (10% goat serum in PBS) and incubated with the primary antibodies mouse anti-human α-SMA (cat. n°: A5228; Sigma), and rabbit anti-human Col type I antibody (cat. n°: PA1-26204; Affinity Bioreagents) overnight at 4°C, followed by secondary antibody anti- mouse/rabbit rhodamine/FITC- (1∶100, Molecular Probes). Cells were visualized by epifluorescence microscopy (×200; Nikon eclipse TE200 inverted microscope, Tokyo, Japan). Visible images were capture for each experimental condition to evaluate the change of epithelial phenotype to mesenchymal phenotype.

### Western Blot

Western blot analysis was used to detect changes in p-Smad3, p-ERK1/2 (42–44 kD) and p-Akt as well as in nuclear β-catenin in liver epithelial THLE-3 cells. THLE-3 cells were homogenized and lysed on ice with a lysis buffer consisting of 20 mM Tris, 1 mM ethylenediaminetetraacetic acid (EDTA), 150 mM NaCl, 0.1% Triton X-100, 1 mM dithiothreitol and 1 µg/ml pepstatin A supplemented by a complete protease inhibitor cocktail to extract total protein. Nuclear protein extraction was performed with the nuclear active motif extraction kit (Active Motif Europe, Rixensart, Belgium) according to the manufacturer's protocol. The Bio-Rad assay (Bio-Rad Laboratories Ltd., Herts, UK) was used (following manufacturer's instructions) to quantify the level of protein in each sample to ensure equal protein loading. Sodium dodecyl sulphate polyacrylamide gel electrophoresis was used to separate the proteins according to their molecular weight. Briefly, 10 µg proteins (denatured) along with a molecular weight protein marker, Bio-Rad Kaleidoscope marker (Bio-Rad Laboratories), were loaded onto an acrylamide gel consisting of a 5% acrylamide stacking gel stacked on top of a 10% acrylamide resolving gel and run through the gel by application of 100 V for 1 h. Proteins were transferred from the gel to a polyvinylidene difluoride membrane using a wet blotting method. The membrane was blocked with 5% Marvel in PBS containing 0.1% Tween20 (PBS-T) and then probed with a rabbit anti-human phospho-Smad3 (cat. n°: PS1023; Calbiochem), mouse anti-human phospho-ERK1/2 (cat. n°: M-9692; Sigma), rabbit anti-human pAkt antibody (Thr308; cat. n°: 2965; Cell Signalling), and rabbit anti-human β-catenin antibody (cat n^o^. NBP1-89989, Novus Biologicals, Cambridge, UK;), and normalised to total rabbit anti-human ERK1/2 antibody (catalogue no. 4695, Cell Signalling, Boston, Massachusetts, USA), total rabbit anti-human Smad3 (cat n°: 566414; Calbiochem), total rabbit anti-human Akt (cat. n°: 4691; Cell Signalling) or total lamin A (polyclonal antibody cat n°: L1293; Sigma) for nuclear protein expression as internal standards. The enhanced chemiluminescence method of protein detection using enhanced chemiluminescence reagents, ECL plus (Amersham GE Healthcare, Buckinghamshire, UK), was used to detect labelled proteins. Quantification of protein expression was performed by densitometry relative to total Smad3, ERK1/2, Akt or Lamin A internal controls using the software GeneSnap version 6.08.

### Real Time RT-PCR

Total RNA was isolated from human liver epithelial THLE-3 cells by using TriPure® Isolation Reagent (Roche, Indianapolis, USA). The integrity of the extracted RNA was confirmed with Bioanalizer (Agilent, Palo Alto, CA, USA). The reverse transcription was performed in 300 ng of total RNA with TaqMan reverse transcription reagents kit (Applied Biosystems, Perkin-Elmer Corporation, CA, USA). cDNA was amplified with specific primers and probes predesigned by Applied Biosystems for *α-SMA* (Hs00559403_m1), α_1_(I)-collagen (*Col type I*; cat. n°: Hs00164004_m1), *vimentin* (cat. n°: Hs 00958116_m1), *E-cadherin* (cat. n°: Hs01023894_m1), *zona occludens-1* (*ZO-1*; cat. n°: Hs01551861_m1), *Slug* (cat. n°: Hs00950344_m1), *Snail* (cat. n°: Hs00195591_m1) and *GAPDH* (pre-designed by Applied Biosystems, cat. n°: 4310884E) as a housekeeping in a 7900HT Fast Real-Time PCR System (Applied Biosystem) using Universal Master Mix (Applied Biosystems). Relative quantification of these different transcripts was determined with the 2^−ΔΔCt^ method using GAPDH as endogenous control (Applied Biosystems; 4352339E) and normalized to control group.

### Statistical Analysis

Statistics of results was carried out by parametric analysis. *P*<0.05 was considered statistically significant. Results were expressed as mean ± SD for patient clinical and laboratory data values, and as mean ± SEM of n experiments for *in vitro* studies. Two-group comparisons were analysed using the Student's t-test. Multiple comparisons were analysed by one-way analysis of variance followed by Bonferroni post hoc test.

## Results

### Effect of Omegaven® on Liver Function of Patients with PNALD

**Figure 1:**
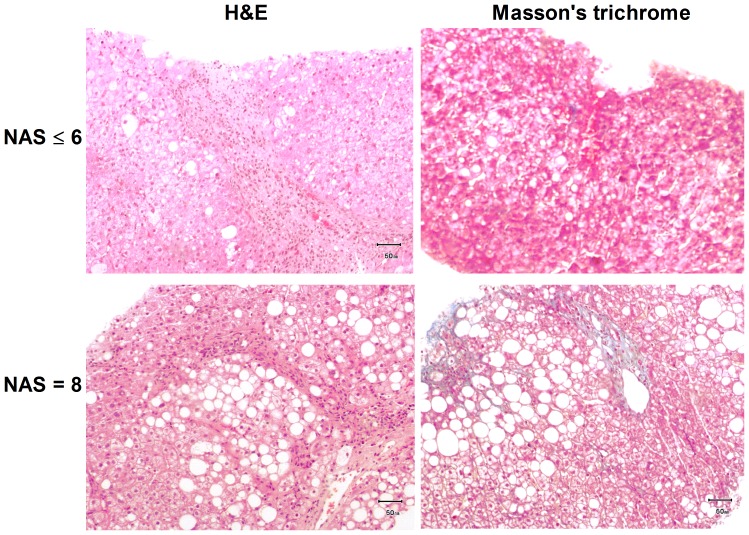
Histological liver representation of parenteral nutrition-associated liver disease patients. Hematoxilin & eosin and masson's trichrome staining of liver biopsies taken for diagnosis purposes. The images are representative of patients with non-alcoholic liver hepatic disease score (NAS) ≤6 and patients with NAS>6 who initiated Omegaven® treatment.

Ten patients with long-term home PN and with non-alcoholic fatty liver disease (NAFLD) were studied. Two patients with the highest baseline NAS score of 8 (see Fig.1 and table 1 for basal characteristics) were selected to start a home PN with Omegaven® as the sole source of PN lipids according to the standard-of-care guidelines. These two patients were with home PN 8 ±1.4 years and with ClinOleic® during the last two years and until the moment of lipid change. Liver biopsies at the moment of Omegaven® infusion showed a high degree of steatohepatitis with inflammatory cell infiltration and a moderate level of fibrosis represented by the blue collagen of masson's trichrome staining (Fig.1). Omegaven® was administered during four months treatment period, followed by SMOFlipid® infusion during the next two months. The other eight home PN patients showed a NAS score ≤6 and received commercial lipid emulsions before and during the study (see Fig. 1 for basal liver biopsy). Four of them were with home PN for 9.5±1.9 years, and initiated ClinOleic® two years before the study. Three patients were under PN 6±1.2 years initiating SMOFlipid® in PN one year before the study. The last patient received Lipofundin® MCT/LCT® in PN during two years and during the study (table 1). Basal demographic, clinical and hepatic score values are showed in table 1.

**Table 1:** Baseline clinical characteristics.

	Omegaven® group (n = 2)	ClinOleic® group (n = 4)	Lipofundin® group (n = 1)	SMOFlipid® (n = 3)
Sex (M/F)	1/1	2/2	0/1	1/2
Age. yr	28.5±0.75	35±23.8	71	48±26
BMI	22.9±0.02	23.3±1.9	23.5	23.4±1.6
Years of HNP	8±1.4	9.5±1.9	2	6±1.2
fibrosis score	F1	F1	F1	F2
Steatosis score	S3	S2	S2	S2
NAS score	8	4	6	5

Clinical data was collected at the moment of inclusion. The Omegaven® group data was collected before starting the 4-mounth Omegaven® course. ClinOleic®, Lipofundin® and SMOFlipid® group data was collected during chronic parenteral nutrition at the moment of the study inclusion.

HNP: home parenteral nutrition; BMI: body mass index; NAS: non-alcoholic liver hepatic disease score.

At baseline, all patients included in this study showed a characteristic increase of hepatic enzymes aspartate transaminase (ASL), alanine transaminase (ALT), γ-glutamyl transpeptidase (GGT) compared to normal laboratory values and healthy subjects (see table 1, table 2 and table 3). Of interest, levels of fibrotic mediators in serum such as matrix metalloproteinase 9 (MMP9) and transforming growth factor beta 1 (TGFβ1) were also significantly elevated compared to those levels of healthy subjects (see table 2 and table 3). In a similar manner, pro-inflammatory and fibrotic cytokines tumor necrosis factor alpha (TNFα), interleukin (IL)-6, IL-1β, IL-8 were significantly increased (Table 2).

**Table 2:** Dynamic laboratory values during home parenteral nutrition (PN).

	Omegaven® (n = 2)					SMOFlipid® (n = 2)	
Serum parameters (reference normal value)	Month 0	Month 1	Month 2	Month 3	Month 4	Month 5	Month 6
AST (11–39 IU/L)	97.5±33.2	38.5±24.7*	38.4±9.1*	30.5±2.1*	27.5±6.3*	31±11.3	69±7*#
ALT (7–33 IU/L	179±70.71	41.5±23.3*	36±7 *	30.5 (7.7) *	30.5 (1.9*)	60.5 (6.3) *#	110±41 *#
GGT (8–55 IU/L)	73.5±65.7	28.5±2.1*	21.5±6.3*	31 (25.4) *	35.5 (19) *	50.5 (40.3)	69.5±31.8 *#
Alkaline phosphatase (50–300 IU/L)	150.5±58.6	116±57.9	98±36.7	115±9.8	113.5±7.7	113.6±7.8	125±19.8
Total bilirubin (<2.5 mg/dL)	0.76±0.05	0.66±0.48	0.75±0.4	0.53±0.01	0.8±0.3	0.8±0.39	0.7±0.03
Prealbumin. (18–45 mg/dL)	20.4±2	22.3±14.5	24.2±5	20.1±0.8	21.4±0.9	20.9±0.8	22.6±4.2
Serum albumin. (3.2–5.2 g/dL)	3.8±0.2	3.6±0.7	3.8±0.2	3.8±0.07	3.8±0.14	3.6±0.07	3.8±0.2
Total protein g/dL	6.9±0.14	7.9±0.21	7.4±0.14	7.2±0.14	7.1±0.21	6.6±1.2	7.3±0.14
Platelets (135–350 cells 10^9^/L)	161±2.8	219±41	180.5±16.2	216±38	176±18.3	176±18.3	196.5±57.2
Hemoglobin (13.5–18 g/dL)	13.7±0.56	12.9±1.76	12.4±0.6	13.1±0.2	13.2±0.1	13.2±0.1	13.2±0.3
Leukocytes (5–10^10^cells/L)	5.3±0.28	6.5±0.28	7.2±0.35	8.3±0.9	5.9±2.3	7.6±1.1	5.5±0.07
Neutrophils %	52.2±13.6	48.1±1.2	51.8±0.7	47.1±3.1	35.8±5*	56±9.4 #	57.6±3.2 #
Lymphcytes %	25.3±0.35	41.5±4.3*	39.2±4.3*	40.9±10.4*	49.3±8.2*	29.3±5.9 #	32.5±0.3 #
Monocytes %	12.1±0.2	9.2±0.1	9.3±0.2	9.1±0.1	9.5±0.3	14.3±1.1	14.2±0.2
MMP-9 (ng/mL)	970±171.4	95.1±69*	107.6±6.1*	113.4±75*	96±26.9*	305.5±99.7*#	564.7±122.3*#
TGFβ1(ng/mL)	42±5.6	13.8±2*	8.8±0.7*	9.3±1.3*	8.7±2*	18.3±1.4*#	32.6±0.67*#
TNFα (ng/mL)	631.7±78.2	122.2±14.2*	110.8±11.9*	107±12*	102.2±14.3*	298.5±31.8*#	449.5±40.3*#
IL1β (ng/mL)	34.8±4.9	7.8±1.5*	8.2±1.8*	7.2±0.7*	6.6±0.4*	11.7±5.2*#	35.3±2.7#
IL-8 (ng/mL)	510.3±72.2	59.6±18.8*	48.1±6*	46.8±10.3*	42.9±4.8*	58.5±13.6*	381.7±43.3*#
IL-6 (ng/mL)	28.3±6	8.5±0.26*	6.3±1.1*	5.8±0.08*	5.1±0.26*	12.5±9.7*#	23.6±2.2#

Two patients with long-term parenteral nutrition associated liver disease and diagnosed of severe non-alcoholic steatohepatitis (NASH), were recruited to start Omegaven® (1 g/kg; 5 PN/week) for four months. After, four months of treatment, parenteral lipid emulsion was changed to SMOFlipid® for another 2 months. *p<0.05 *vs*. values of month 0. #p<0.05 *vs*. values at month 4.

ALP. alkaline phosphatase; ALT. alanine transaminase; AST. aspartate transaminase; GGT. γ-glutamyl transpeptidase. MMP-9.

**Table 3:** Hepatic and inflammatory marker values in healthy subjects, and patients after four months of Omegaven® treatment, and patients with chronic ClinOleic®, Lipofundin® and SMOFlipid® based-home parenteral nutrition. *p<0.05 *vs*. values of healthy and Omegaven®.

	Healthy subjects (n = 6)	Omegaven® (n = 2)	ClinOleic® ((n = 4)	Lipofundin® (n = 1)	SMOFlipid® (n = 3)
AST (11–39 IU/L)	22±4.2	27.5±6.3	83±25.8*	57*	44±9.5*
ALT (7–33 IU/L	19±2.1	30.5±1.9	152.3±52.3*	52*	70.3±17.6*
GGT (8–55 IU/L)	27±6.2	35.5±19	64.7±40.7 *	127*	62±28.6*
Alkaline phosphatase (50–300 IU/L)	69±2.4	113.5±7.7	140.5±36.4*	111	127.3±24.5
Total bilirubin (<2.5 mg/dL)	0.5±0.1	0.8±0.3	0.6±0.12	0.9	0.6±0.4
Prealbumin. (18–45 mg/dL)	22.4±0.3	21.4±0.9	20±1.5	18	21.9±2.2
Serum albumin. (3.2–5.2 g/dL)	4.1±0.1	3.8±0.14	3.8±0.15	3.5	3.5±0.15
Total protein. g/dL	7.1±1.1	7.1±0.21	6.7±0.2	7	6.6±0.8
Platelets (135–350 cells 10^9^/L)	213±69	176±18.3	162.3±9.6	104	217±72
Hemoglobin (13.5–18 g/dL)	16.2±1.3	13.2±0.1	13.8±0.4	12.5	13.5±0.6
Leukocytes (5–10^10^cells/L)	6.5±0.2	5.9±2.3	6±0.8	4.7	9.3±3.4
Neutrophils %	52±3.1	35.8±5*	58.6±10.7	68	61.3±11.3
Lymphcytes %	34±0.1	49.3±8.2*	24.7±0.8	18	24±10.1
Monocytes %	7.2±0.4	9.5±0.3	15.2±0.4	16.3	14.6±0.1
MMP9 (pg/mL)	112.1±19.3	96±26.9	825.5±214.6*	476.7*	771.2±367.9*
TGFβ1(ng/mL)	11.2±1.1	8.7±2	40.1±11.8*	34.3*	34.8±3.8*
TNFα (pg/mL)	138.4±13.4	102.2±14.3	526.6±143.8*	598.1*	513.8±114.9*
IL1β (pg/mL)	7.1±1.2	6.6±0.4	28.2±13.4*	41.2*	38.1±5.2*
IL-8 (pg/mL)	41.6±3.2	42.9±4.8	443.8±94.5*	491.2*	356.6±53.1*
IL-6 (pg/mL)	7.4±0.6	5.1±0.26	25.6±5.6*	37.1*	23.1±1.7*

At the first month of starting Omegaven® infusion, the hepatic enzymes decreased to laboratory normal values, and the serum fibrotic mediators as well as the inflammatory cytokines were significantly reduced to levels comparable to those observed for healthy subjects (see table 2 and table 3). Hepatic enzymes, serum fibrotic mediators and cytokines remained diminished during the four months of Omegaven® parenteral infusion. After Omegaven® treatment, SMOFlipid® was infused for a period of two months. Serum hepatic enzymes, fibrotic mediators and cytokines increased again, and returned to similar levels observed before Omegaven® treatment (table 2).

Interestingly, Omegaven® infusion reduced the number of blood neutrophils and monocytes and increased the number of lymphocytes. Again, SMOFlipid® infusion returned to initial levels the number of neutrophils, monocytes and lymphocytes to those observed before Omegaven® treatment.

### Effects of Different Parenteral Lipid Emulsions on the LPS-Induced Inflammatory and Pro-fibrotic Protein Release in Monocytes from Healthy Subjects

**Figure 2:**
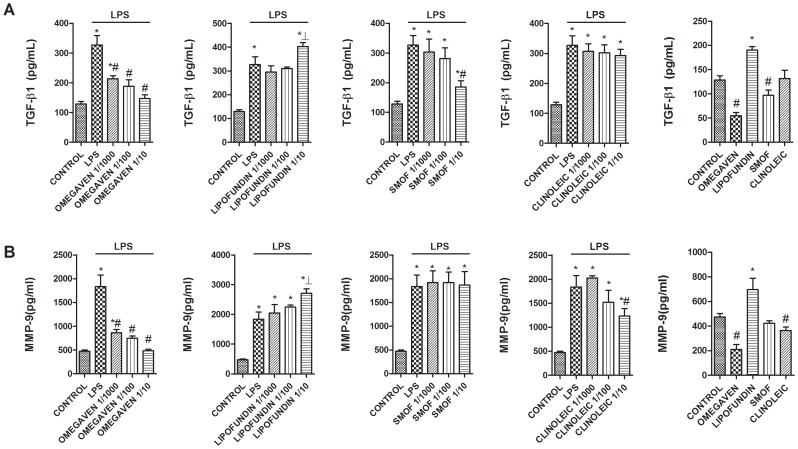
Effect of different commercial parenteral lipid emulsions on Lipopolysaccharide (LPS)-induced TGFβ1 and MMP-9 secretion in human monocytes. Human monocytes were isolated from healthy subjects and incubated in presence or absence of lipid emulsions Omegaven® 10%, Lipofundin MCT/LCT® 20%, ClinOleic® 20% or SMOFlipid® 20% at different dilutions, for 30 min followed by LPS 1 µg/mL stimulation for additional 24 hours. (A) TGFβ1 and (B) MMP-9 were measured in cell culture supernatants. The effect of lipid emulsions without stimulus was tested at 1/10 dilution. Results are expressed as means ± SEM of six independent experiments. *p<0.05 related to the control group. #*p*<0.05 values below stimulus; ⊥*p*<0.05 values above the stimulus.

**Figure 3:**
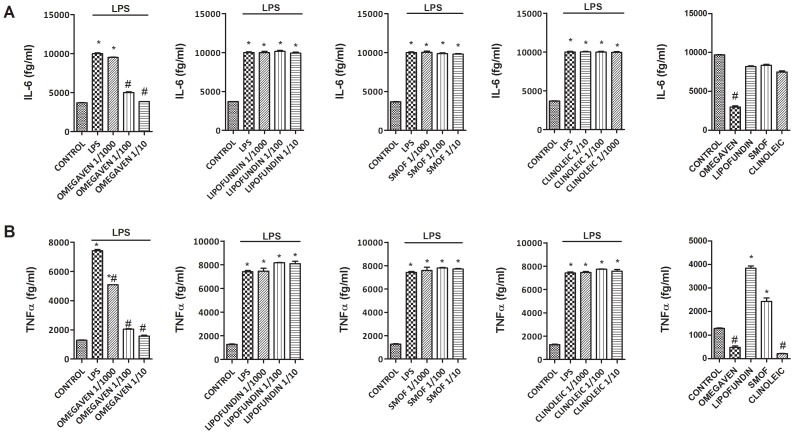
Effect of different commercial parenteral lipid emulsions on Lipopolysaccharide (LPS)-induced IL-6 and TNFα secretion in human monocytes. Human monocytes were isolated from healthy subjects and incubated in presence or absence of lipid emulsions Omegaven® 10%, Lipofundin MCT/LCT® 20%, ClinOleic® 20% or SMOFlipid® 20% at different dilutions, for 30 min followed by LPS 1 µg/mL stimulation for additional 24 hours. (A) IL-6 and (B) TNFα were measured in cell culture supernatants. The effect of lipid emulsions without stimulus was tested at 1/10 dilution. Results are expressed as means ± SEM of six independent experiments. *p<0.05 related to the control group. #*p*<0.05 values below stimulus; ⊥*p*<0.05 values above the stimulus.

**Figure 4:**
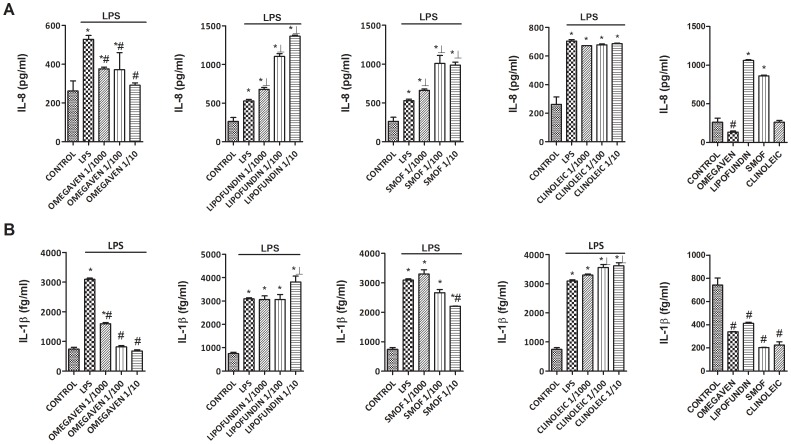
Effect of different commercial parenteral lipid emulsions on Lipopolysaccharide (LPS)-induced IL-8 and IL-1β secretion in human monocytes. Human monocytes were isolated from healthy subjects and incubated in presence or absence of lipid emulsions Omegaven® 10%, Lipofundin MCT/LCT® 20%, ClinOleic® 20% or SMOFlipid® 20% at different dilutions, for 30 min followed by LPS 1 µg/mL stimulation for additional 24 hours. (A) IL-8 and (B) IL-1β were measured in cell culture supernatants. The effect of lipid emulsions without stimulus was tested at 1/10 dilution. Results are expressed as means ± SEM of six independent experiments. One-way ANOVA was followed by the post hoc Bonferroni test. *p<0.05 related to the control group. #*p*<0.05 values below stimulus; ⊥*p*<0.05 values above the stimulus.

**Figure 5:**
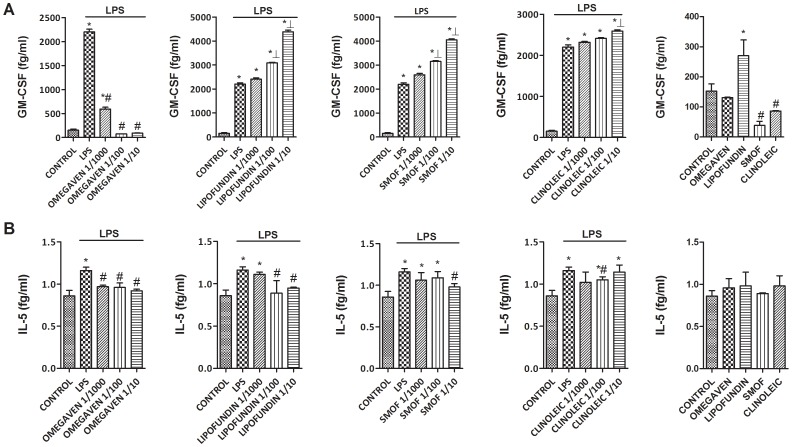
Effect of different commercial parenteral lipid emulsions on Lipopolysaccharide (LPS)-induced GM-CSF and IL-5 secretion in human monocytes. Human monocytes were isolated from healthy subjects and incubated in presence or absence of lipid emulsions Omegaven® 10%, Lipofundin MCT/LCT® 20%, ClinOleic® 20% or SMOFlipid® 20% at different dilutions, for 30 min followed by LPS 1 µg/mL stimulation for additional 24 hours. (A) GM-CSF and (B) IL-5 were measured in cell culture supernatants. The effect of lipid emulsions without stimulus was tested at 1/10 dilution. Results are expressed as means ± SEM of six independent experiments. One-way ANOVA was followed by the post hoc Bonferroni test. *p<0.05 related to the control group. #*p*<0.05 values below stimulus; ⊥*p*<0.05 values above the stimulus.

**Figure 6:**
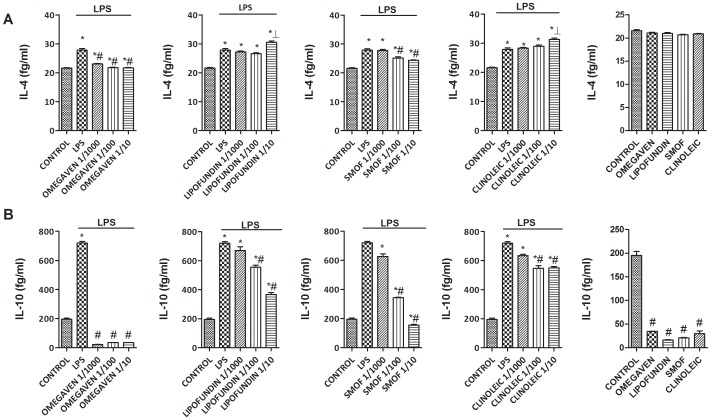
Effect of different commercial parenteral lipid emulsions on Lipopolysaccharide (LPS)-induced IL-4 and IL-10 secretion in human monocytes. Human monocytes were isolated from healthy subjects and incubated in presence or absence of lipid emulsions Omegaven® 10%, Lipofundin MCT/LCT® 20%, ClinOleic® 20% or SMOFlipid® 20% at different dilutions, for 30 min followed by LPS 1 µg/mL stimulation for additional 24 hours. (A) IL-4 and (B) IL-10 were measured in cell culture supernatants. The effect of lipid emulsions without stimulus was tested at 1/10 dilution. Results are expressed as means ± SEM of six independent experiments. One-way ANOVA was followed by the post hoc Bonferroni test. *p<0.05 related to the control group. #*p*<0.05 values below stimulus; ⊥*p*<0.05 values above the stimulus.

**Figure 7:**
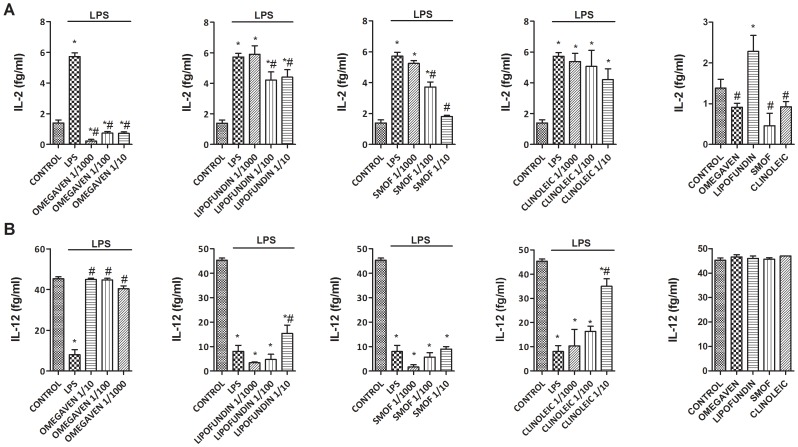
Effect of different commercial parenteral lipid emulsions on Lipopolysaccharide (LPS)-induced IL-2 and IL-12 secretion in human monocytes. Human monocytes were isolated from healthy subjects and incubated in presence or absence of lipid emulsions Omegaven® 10%, Lipofundin MCT/LCT® 20%, ClinOleic® 20% or SMOFlipid® 20% at different dilutions, for 30 min followed by LPS 1 µg/mL stimulation for additional 24 hours. (A) IL-2 and (B) IL-12 were measured in cell culture supernatants. The effect of lipid emulsions without stimulus was tested at 1/10 dilution. Results are expressed as means ± SEM of six independent experiments. One-way ANOVA was followed by the post hoc Bonferroni test. *p<0.05 related to the control group. #*p*<0.05 values below stimulus; ⊥*p*<0.05 values above the stimulus.

Omegaven® (1/10-1/1000) dose-dependently inhibited the LPS-induced TGFβ1 and MMP-9 release (Figs. 2A and 2B) and decreased basal TGFβ1 and MMP-9 secretion in human monocytes. Conversely, Lipofundin MCT/LCT® (1/10-1/1000) dose-dependently increased TGFβ1 and MMP-9 secretion induced by LPS and increased the basal release *per se* (Figs. 2A and 2B). SMOFlipid® slightly reduced TGFβ1 secretion at 1/10 dilution whereas ClinOleic® lipid emulsion attenuated only MMP-9 release at 1/10 concentration (Figs. 2A and 2B). In other experiments, Omegaven® (1/10-1/1000) dose-dependently inhibited the release of the pro-inflammatory cytokines IL-6, TNFα (Figs. 3A and 3B), IL-8, IL-1β (Fig. 4) as well as the T_H_2 cytokines GM-CSF, IL-5 and IL-4 (Figs. 5 and 6) induced by LPS. Lipofundin MCT/LCT® (1/10-1/1000) showed a pro-inflammatory profile elevating even more the LPS-induced IL-8, IL-1β (Fig. 4A and 4B), GM-CSF (Fig. 5A) and IL-4 (Fig. 6A) without affecting IL-6 or TNFα (Fig. 3A and 3B). Furthermore, cell exposure to Lipofundin MCT/LCT® (1/10) without stimulus also showed a pro-inflammatory profile, increasing significantly TNFα, IL-8 and GM-CSF release. ClinOleic® (1/10-1/1000) did not modify the increase of pro-inflammatory cytokines, with the exception of a small increase of IL-1β, GM-CSF and IL-4. SMOFlipid® (1/10-1/1000) showed a variable profile, increasing the LPS-induced IL-8 and GM-CSF release and inhibiting the secretion of IL-1β and IL-4. Finally, the stimulation of monocytes with LPS increased the release of T_H_1 cytokines IL-10 and IL-2 while downregulated the secretion of IL-12. Omegaven® (1/10-1/1000) suppressed the IL-10 and IL-2 release and increased IL-12 to control levels (Fig. 6 and 7). Lipofundin MCT/LCT®, SMOFlipid® and ClinOleic® dose-dependently inhibited the increase of IL-10 and IL-2 release induced by LPS, and only ClinOleic® at 1/10 concentration elevated IL-12 to control levels (Figs. 6 and 7).

### Omegaven® Inhibits Liver Epithelial to Mesenchymal/Myofibroblast Transformation Induced by TGFβ1

**Figure 8:**
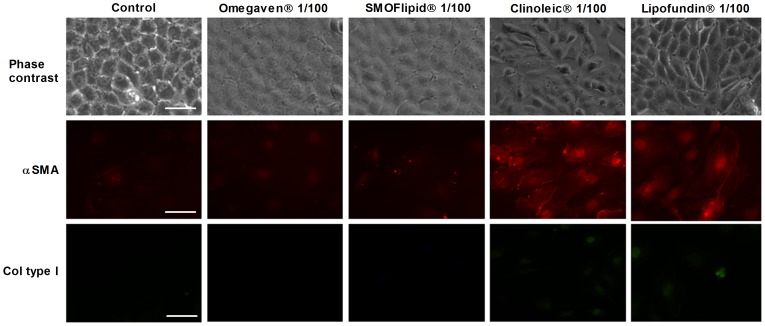
Effect of different lipid emulsions on the expression of myofibroblast markers. Human liver epithelial cell line THLE-3 was incubated in presence or absence of lipid emulsions Omegaven® 10%, Lipofundin MCT/LCT® 20%, ClinOleic® 20% or SMOFlipid® 20% at 1/100 dilutions for 72 hours. Representative visible morphology and immunofluorescences for alpha smooth muscle actin (αSMA) and collagen type I (col type I) are showed.

**Figure 9:**
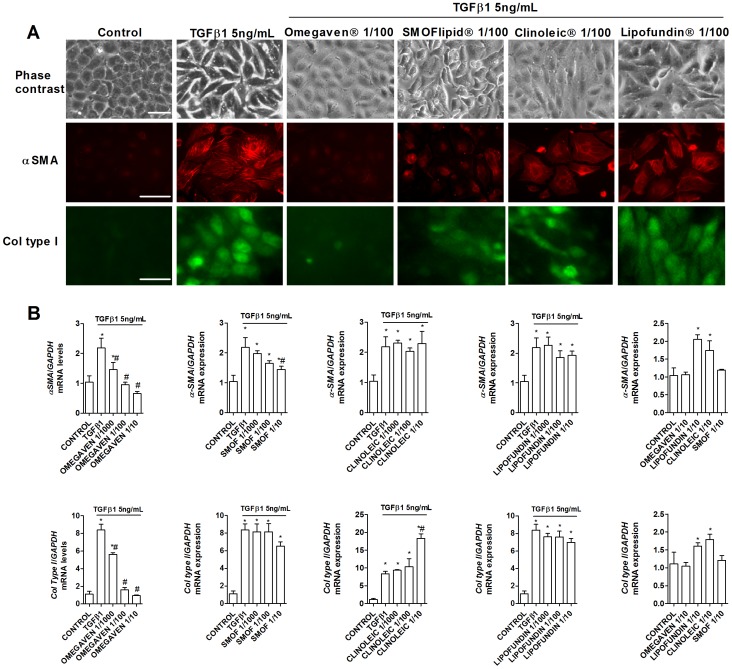
Omegaven® inhibits myofibroblast markers induced by TGFβ1. Human liver epithelial cell line THLE-3 was incubated in presence or absence of lipid emulsions Omegaven® 10%, Lipofundin MCT/LCT® 20%, ClinOleic® 20% or SMOFlipid® 20% at different dilutions, for 30 min followed by TGFβ1 5 ng/mL stimulation for additional 72 hours. (A) Visible morphology and immunofluorescence for alpha smooth muscle actin (αSMA) and collagen type I (col type I) distribution and expression. B) Expression of mRNA of *αSMA* and *col type I*. Scale bar: 10 µm. Results are expressed as means ± SEM of six independent experiments. *p<0.05 related to the control group. #*p*<0.05 related to the stimulus.

**Figure 10:**
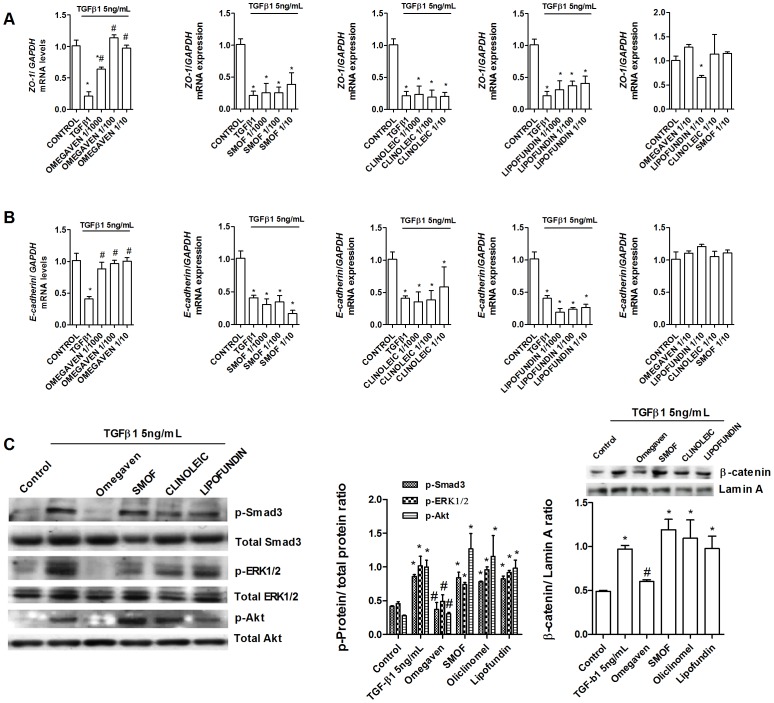
Omegaven® inhibits epithelial to mesenchymal transition induced by TGFβ1. Human liver epithelial cell line THLE-3 was incubated in presence or absence of lipid emulsions Omegaven® 10%, Lipofundin MCT/LCT® 20%, ClinOleic® 20% or SMOFlipid® 20% at different dilutions, for 30 min followed by TGFβ1 5 ng/mL stimulation for additional 72 hours (A and B) or 25 min (C). (A) Expression of mRNA of *ZO-1* and (B) *E-cadherin*. (C) Phosphorylation of Samd3, ERK1/2 and Akt and nuclear expression of β-catenin. Representative western blot are showed and quantified in graphic bars. Results are expressed as means ± SEM of six independent experiments. *p<0.05 related to the control group. #*p*<0.05 related to the stimulus.

**Figure 11:**
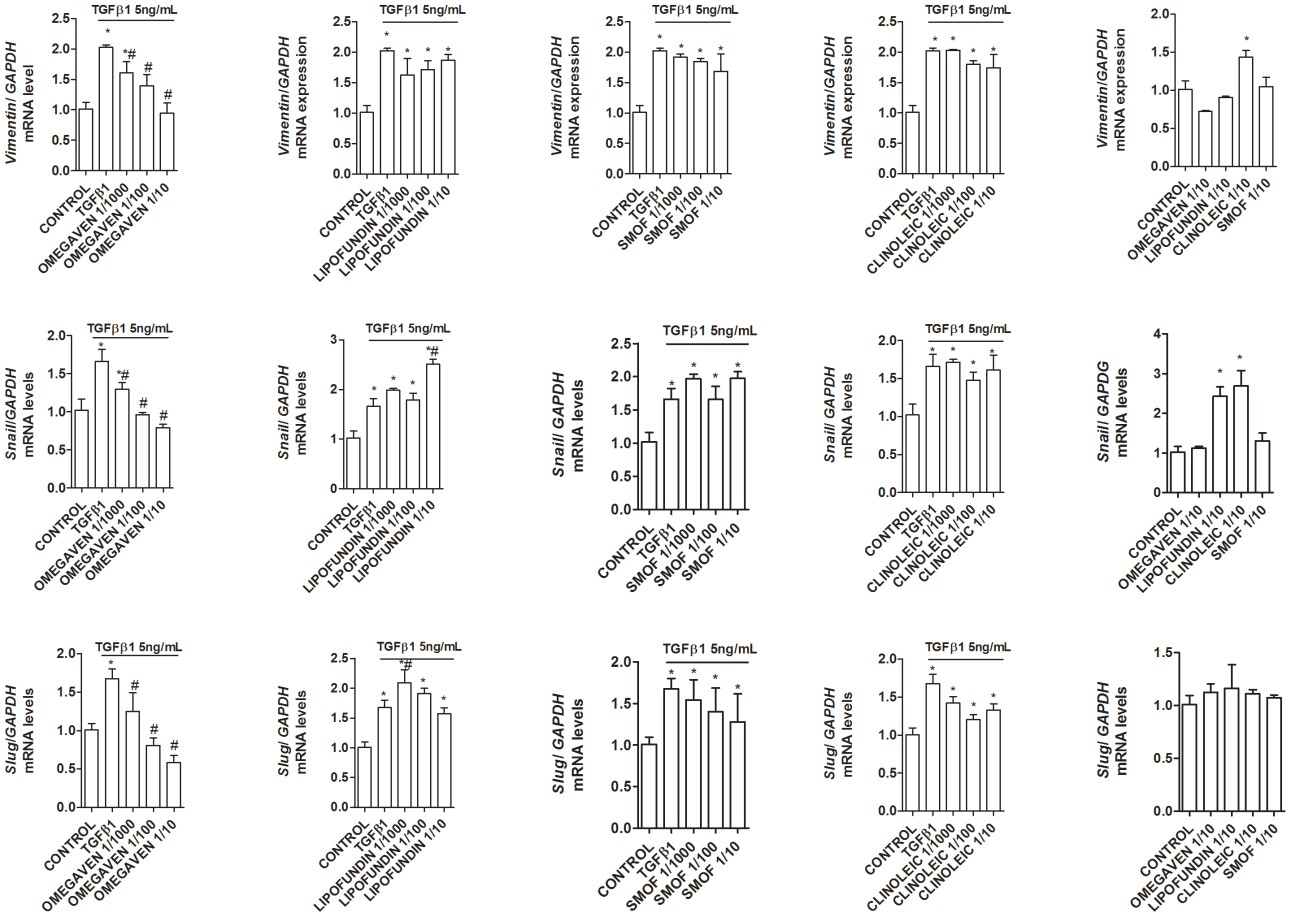
Omegaven® inhibits transcription factor an markers related with epithelial to mesenchymal transition induced by TGFβ1. Human liver epithelial cell line THLE-3 was incubated in presence or absence of lipid emulsions Omegaven® 10%, Lipofundin MCT/LCT® 20%, ClinOleic® 20% or SMOFlipid® 20% at different dilutions, for 30 min followed by TGFβ1 5 ng/mL stimulation for additional 72 hours. Expression of mRNA of *Snail, Slug*, and vimentin. Results are expressed as means ± SEM of six independent experiments. One-way ANOVA was followed by the post hoc Bonferroni test. *p<0.05 related to the control group. #*p*<0.05 related to the stimulus.

The liver epithelial THLE-3 cell line cultured in the absence of TGFβ1 maintained classic cobblestone epithelial morphology as assessed by phase contrast light microscopy (Fig. 8). Liver epithelial cells incubated with ClinOleic® 1/100 and Lipofundin MCT/LCT® 1/100 significantly increased the myofibroblast-like phenotype and markers alpha smooth muscle cell (αSMA) and collagen type I (Col Type I) (Fig. 8 and 9B), whilst Omegaven® 1/100 and SMOFlipid® 1/100 did not modify the epithelial phenotype. A concentration of 5 ng/mL of TGFβ1 (72 h) began to induce morphological changes in liver epithelial cells, characterized by a more mesenchymal fibroblast-like morphology with reduced cell-cell contact (Fig. 9A). Omegaven® (1/100) but not Lipofundin MCT/LCT®, SMOFlipid® or ClinOleic®, preserved the epithelial phenotype. To further study the epithelial to mesenchymal (EMT) process, it was analyzed the distribution and expression of alpha smooth muscle cell (αSMA) and collagen type I (Col Type I) by immunofluorescence. TGFβ1 produced *de novo* formation of αSMA fibers and Col Type I that were inhibited by Omegaven® (1/100) but not by Lipofundin MCT/LCT®, SMOFlipid® and ClinOleic® (Fig. 9A). Similar results were observed at the level of gene expression. Omegaven® and in a lesser extent SMOFlipid® dose-dependently inhibited the increase of *αSMA* and *Col Type I* gene expression induced by TGFβ1 (Fig. 9B). In contrast, Lipofundin MCT/LCT® and ClinOleic® increased the *αSMA* and *Col Type I* gene expression *per se* and in cells stimulated with TGFβ1 (Fig. 9B). According to these observations, TGFβ1 significantly decreased the expression of epithelial markers *E-cadherin* and *ZO-1*, both tight and adherent junctions implicated in the epithelial barrier conformation (Figs. 10A and B). Unlike other lipid emulsions studied, Omegaven® was able to increase *E-cadherin* and *ZO-1* expression to control levels (Figs. 10A and B). In other experiments, only Omegaven® was able to decrease vimentin expression as well as mesenchymal transcription factors *Snail* and *Slug* increased by TGFβ1 (Fig. 11).

Mechanistically, Omegaven® inhibited the phosphorylation of Smad3, ERK1/2 and Akt as well as the nuclear translocation of β-catenin induced by TGFβ1, all of them implicated in the EMT process [19].

## Discussion

The present manuscript provides a novel explanation of the mechanisms involved in the effect of the Fish oil/ω-3 PUFAs reversing long-term home PNALD.

This study shows a proof of concept evidence in which 100% fish oil Omegaven® lipid treatment restores the hepatic enzymes, fibrotic and inflammatory cytokine markers to healthy normal values. *In vivo* results were further corroborated on *in vitro* human monocytes, where Omegaven® product showed strong anti-inflammatory properties related with a broad spectrum of innate and adaptative T_H_1 and T_H_2 responses as well as an inhibitory effect on profibrotic TGFβ1 and MMP-9 secretion. Finally, unlike parenteral lipid emulsions based in soybean oil, Omegaven® inhibited the mesenchymal/myofibroblast transformation of liver epithelial cells which overall suggest an anti-inflammatory and antifibrotic profile for this ω-3 fatty acid emulsion, potentially useful in those situations of adult liver disease secondary to long-term PN.

In contrast to children PNALD, liver complications associated with long-term PN in adults are characterized by the presence of advanced non-alcoholic steatohepatitis (NASH) which may progress to fibrosis or cirrhosis [11]. In this work, all patients recruited showed mild to severe NASH with a nascent degree of fibrosis (F1-F2) and absence of cholestasis or cholelitiasis that characterize the pediatric population. The Omegaven® lipid emulsion in PN normalized the hepatic enzymes, inflammatory cytokines and profibrotic markers at the first month of treatment. To our knowledge, only a few case-reports have been published regarding the role of ω-3 PUFA-rich, oil-based lipid emulsions in the treatment of the adult long-term PNALD [20], [21], [22]. Venecourt, et al. [21] described an adult patient with long-term PNALD with abnormal liver function tests including jaundice, who improved rapidly upon changing a soybean/olive oil-based PN to one based on pure fish oil ω-3 fatty acids. Burns et al. [20] described an adult home PN patient where the use of a fish oil-based formula appeared to reverse PNALD. Xu, et al reported that ω-3/ω-6 mixed PUFAs reverse biopsy-proven short term PNALD in adults and concluded that parenteral ω-3 PUFA-enriched fat emulsions are beneficial and safe in the management of PNALD in adults [22]. However, other authors reported that treatment with ω-3 fatty acids did not completely reverse liver fibrosis in children [23], [24]. ω-3 PUFAs also can exert some deleterious effects such as antiplatelet action with risk for bleeding, thus some authors purpose a well-balanced mixture of ω-3, ω-6, and ω-9 fatty acids to compensate these effects.

In this work, we showed that, once hepatic function and systemic inflammation was controlled, reintroduction of a mixture of ω-6/ω-3/ω-9/MCT fatty acid emulsion SMOFlipid® increased again the liver enzymes and inflammatory and pro-fibrotic markers in serum to similar levels to those observed before starting Omegaven® infusion. These observations suggest that long-term PNALD may require either a large period of treatment (more than 4 months) or a definitive change on the relation of the fatty acids profile in favor of ω-3 PUFAs. However, since Omegaven® does not provide the required essential fatty acid load for optimal nutrition, the ideal situation for long-term parenteral nutrition-associated liver disease in adults would be to alternate 2-4 months of parenteral nutrition with Omegaven® lipids with 2 months of an equilibrate fish oil SMOFlipid® in repetitive cycles.

But other factors cannot be excluded. Differences between α-tocopherol and phytosterol content between different commercial lipid emulsions are marked, and they may, in part, influence either the development of hepatic lesions or the resolution of the PNALD [25].

Interestingly, we observed that innate immune cells such as neutrophils and monocytes decreased in serum while adaptative lymphocytes increased during Omegaven® infusion. Similar findings were also observed by Burns et al. [20] after 16 weeks of Omegaven® treatment. This observation could explain, almost in part, the decrease of IL-6, IL-8, IL-1β and TNFα pro-inflammatory cytokines and the pro-fibrotic TGFβ1 and MMP-9 in serum, as neutrophils and monocytes are primed to secrete high amounts of these molecular profile in chronic inflammatory conditions [26].

However, due to the small sample size analyzed in this work, it seems premature to extract solid conclusions which represent a limitation of this work; so, future multicentre studies are guaranteed since long term PNALD in adults is not a common disorder.

Monocyte-derived macrophage hepatic infiltration has been proposed as an external source of macrophages in the injured liver. In this regard, inhibition of monocyte liver infiltration in CD11b-DTR transgenic mice, ameliorated liver fibrosis similar to the abrogation of chemokine pathways that control monocyte influx [27].

In this work we observed a strong anti-inflammatory effect of Omegaven® on human monocytes activated by LPS, nearly suppressing IL-6, IL-8, IL-1β and TNFα whose elevated levels have been associated previously with NASH and liver fibrosis [28]. In contrast, the soybean oil-based PN Lipofundin® and, in a lesser extent ClinOleic® and SMOFlipid® did not reduce, but increased the release of pro-inflammatory cytokines. Similar findings were observed for TGFβ1 and MMP-9. While TGFβ1 is increased in serum of patients with NASH and contributes to liver fibrosis secondary to NASH [28], [29], the role of MMP-9 could apparently have a contradictory role. Secretion of MMP-9 allows blood neutrophils and monocytes to infiltrate into the injury liver by means of extracellular cell matrix degradation, thus contributing to inflammatory cell infiltration [30]. By contrast, MMP-9 can be release by liver macrophage Ly-6C^lo^ phenotype that is involved in matrix degradation and thereby favor resolution of liver injury and fibrosis [31]. In the context of our *in vivo* and *in vitro* observations, the reduction of TGFβ1 and MMP-9 in serum and in stimulated monocytes could be related with reduced monocyte liver infiltration and development of liver fibrosis as previously stated [28], [32].

Different liver ‘insult’ damage hepatocytes, which leads to the release of inflammatory mediators. Leukocytes that are recruited to the site of injury phagocytose dead or apoptotic cells and amplify the inflammatory response by generating oxidative stress and pro-inflammatory cytokines, such as TNFα, IL-6 and IL-1β, and by recruiting T cells [33]. Pro-inflammatory mediators and oxidative stress that are generated by cellular damage and inflammatory cells, as well as growth factors and cytokines including platelet-derived growth factor (PDGF) and TGFβ generated by the activation of nuclear enzymes such as Poly (ADP-ribose) polymerase1 (PARP-1) [34] in the damaged liver activate mesenchymal precursor cells in tissues and induce their transdifferentiation to myofibroblasts. TGFβ is the major pro-fibrogenic cytokine and it upregulates αSMA and type I collagen synthesis by hepatic stellate cell and hepatocyte-derived myofibroblasts [18], [35], [36], whereas PDGF induces the proliferation of myofibroblasts.

Respect why ω-3 and ω-6 PUFAs have opposite roles on liver inflammation, different explanations have been provided. Thus, ω-6 PUFA metabolism predominantly results in production of 2-series prostaglandins and thromboxanes and the 4-series leukotrienes [37]. These mediators result in the release of proinflammatory cytokines such as IL-6, initiating leukocyte activation and chemotaxis. The ω3-PUFAs produce 3-series prostaglandins and thromboxanes and the 5-series leukotrienes with immunomodulatory properties. In fact, ω3-PUFAs modulate inflammation through direct effects on transcription regulation. ω3-EPA, DHA, and the derived eicosanoids have been shown to bind PPARs α and γ, and EPA and DHA to bind to G-protein coupled receptors (GPR) 120 and 40. In this regard, recently it has been shown that Omegaven® mediates its anti-inflammatory effects through the stimulation of GPR120 receptor in liver macrophages, promoting the balance to M2 macrophage pro-resolving phenotype [38].

In addition to its anti-inflammatory properties, animal and human liver biopsies suggest that Omegaven® could have a direct liver anti-fibrotic role. Pro-inflammatory mediators that are generated by cellular damage and stimulated immune cells, as well as growth factors and cytokines, activate undiferentiated cells in tissues and induce their transdifferentiation to myofibroblasts, thus initiating and enhancing liver fibrosis [31]. The liver epithelial to mesenchymal/myofibroblast transition (EMT) has been recently emerged as a relevant source of liver myofibroblasts in liver fibrosis. The accumulating evidence has suggested that the EMT contributes to liver fibrosis, similar to processes that occur in other organs, such as the lungs, kidneys, and intestines [19]. An EMT can be found in response to growth factors, such as TGF-β in rat fetal liver cells [39]. Convincing evidence has shown that TGF-β can induce EMT in mouse hepatocytes *in vitro*, thus showing a loss of epithelial markers (Ecadherin, ZO-1) and gain mesenchymal markers (vimentin, αSMA, col type I, FSP1 and β-catenin) [19]. Further evidence has demonstrated, using AlbCre R26RstoplacZ double transgenic mice *in vivo*, that hepatocytes can undergo EMT [36]. In this work, we observed an induction of the mesenchymal/myofibroblast phenotype following chronic TGFβ1 exposure in human hepatocytes. As confirmation of the Omegaven® anti-fibrotic hypothesis, we showed a full inhibitory process of Omegaven® on the TGFβ1-induced EMT process. In contrast, soybean oil based lipid emulsions did not inhibited EMT, and surprisingly increased myofibroblast markers such as vimentin, αSMA and col type I. The molecular approach revealed that Omegaven® is able to inhibit the increase of TGFβ1-induced Smad3, ERK1/2 and Akt phosphorylation as part of the EMT process previously described in other systems [40]. Furthermore, Omegaven® also reduced the expression of the transcription factors Snail and Slug as well as the β-catenin nuclear translocation, all of them implicated in the downregulation of epithelial and upregulation of mesenchymal protein expression [19]. Because Omegaven® lipid emulsion has multiple components, it would be difficult to answer what of the fatty acids composition [41] is the responsible of the anti-EMT effect. Since, the main fatty acids of Omegaven® are EPA and DHA (main responsible of the anti-inflammatory properties), it seems reasonable to think that they are also responsible of the inhibition of myofibroblast formation. In this regard, a recent manuscript showed that DHA, but not EPA, inhibited prostate fibroblast to myofibroblast transition as well as EMT [42], suggesting that Omegaven® DHA fatty acid could be the responsible of the potential anti-fibrotic effects of Omegaven®.

In conclusion, we provide *in vivo* evidence of the inhibitory effect of Omegaven® on inflammatory and pro-fibrotic markers in adult patients with long-term home PNALD which may be potentially useful to those patients with advanced steatohepatitis and liver fibrosis. *In vitro* approaches on human monocytes showed potent anti-inflammatory properties for Omegaven® on a broad spectrum of cytokines and growth factor previously related with liver injury and fibrosis. In addition, Omegaven® inhibited the EMT process in liver epithelial cells as a hallmark of *in vitro* fibrogenesis model. Overall, results provided in this study may be of potential value to justify the use of fish oil lipid emulsions in the PN of adults with long-term home PNALD.

## Supporting Information

S1 Checklist **STROBE Checklist.**
(DOCX)
