# Supplementary material for: Anti-Inflammatory and Anti-Fibrotic Profile of Fish Oil Emulsions Used in Parenteral Nutrition-Associated Liver Disease
Source: PLoS One. 2014 Dec 12;9(12):e115404. doi: 10.1371/journal.pone.0115404 (PMC4264955; doi:10.1371/journal.pone.0115404)
Supplement: S1 Checklist — STROBE Checklist. (DOCX) [file pone.0115404.s001.docx]

STROBE Statement for the manuscript entitled: **Anti-inflammatory and anti-fibrotic profile of fish oil emulsions used in parenteral nutrition-associated liver disease**

**To be published in PLOSONE**

STROBE Statement—checklist of items that should be included in reports of observational studies

|  | Item No. | Recommendation | Page  No. | Relevant text from manuscript |
| --- | --- | --- | --- | --- |
| **Title and abstract** | 1 | (*a*) Indicate the study’s design with a commonly used term in the title or the abstract | 1 | See title in page 1 |
|  |  | (*b*) Provide in the abstract an informative and balanced summary of what was done and what was found | 2 | See abstract in page 2 |
| Introduction | | | |  |
| Background/rationale | 2 | Explain the scientific background and rationale for the investigation being reported | 3-5 | See introduction in page 3 |
| Objectives | 3 | State specific objectives, including any prespecified hypotheses | 5 | See introduction in page 5 |
| Methods | | | |  |
| Study design | 4 | Present key elements of study design early in the paper | Page 5 | Methods: page 5, lines 128-132 |
| Setting | 5 | Describe the setting, locations, and relevant dates, including periods of recruitment, exposure, follow-up, and data collection | Page 6 | Methods: page 6, lines 142-151 |
| Participants | 6 | (*a*) *Cohort study*—Give the eligibility criteria, and the sources and methods of selection of participants. Describe methods of follow-up | Page 6 | Methods: page 6, lines 130-141 |
|  |  | (*b*) *Cohort study*—For matched studies, give matching criteria and number of exposed and unexposed | Not applicable |  |
| Variables | 7 | Clearly define all outcomes, exposures, predictors, potential confounders, and effect modifiers. Give diagnostic criteria, if applicable | Page 5-7 | Methods: lines 133-181 |
| Data sources/ measurement | 8* | For each variable of interest, give sources of data and details of methods of assessment (measurement). Describe comparability of assessment methods if there is more than one group | Page 5-7 lines 142-181 and page 12 lines 300-305 |  |
| Bias | 9 | Describe any efforts to address potential sources of bias | Not applicable |  |
| Study size | 10 | Explain how the study size was arrived at | Not applicable (see page 5 lines 130-131) |  |

| Quantitative variables | 11 | Explain how quantitative variables were handled in the analyses. If applicable, describe which groupings were chosen and why | Page 6, lines 142-151 | Page 12 lines 300-305 |
| --- | --- | --- | --- | --- |
| Statistical methods | 12 | (*a*) Describe all statistical methods, including those used to control for confounding | Page 12 lines 300-305 |  |
|  |  | (*b*) Describe any methods used to examine subgroups and interactions | Not applicable |  |
|  |  | (*c*) Explain how missing data were addressed | Not applicable |  |
|  |  | (*d*) *Cohort study*—If applicable, explain how loss to follow-up was addressed | Not applicable |  |
|  |  | (*e*) Describe any sensitivity analyses | Not applicable |  |
| Results | | | | |
| Participants | 13* | (a) Report numbers of individuals at each stage of study—eg numbers potentially eligible, examined for eligibility, confirmed eligible, included in the study, completing follow-up, and analysed | Page 12, lines 307-317 |  |
|  |  | (b) Give reasons for non-participation at each stage | Not applicable |  |
|  |  | (c) Consider use of a flow diagram | Not applicable |  |
| Descriptive data | 14* | (a) Give characteristics of study participants (eg demographic, clinical, social) and information on exposures and potential confounders |  |  |
|  |  | (b) Indicate number of participants with missing data for each variable of interest | Page 12, lines 307-317 | Table 1 |
|  |  | (c) *Cohort study*—Summarise follow-up time (eg, average and total amount) | Page 12, lines 307-317 |  |
| Outcome data | 15* | *Cohort study*—Report numbers of outcome events or summary measures over time | Page 12-13 lines 307-342 | Table 1, 2 and 3 |
|  |  |  |  |  |
|  |  |  |  |  |
| Main results | 16 | (*a*) Give unadjusted estimates and, if applicable, confounder-adjusted estimates and their precision (eg, 95% confidence interval). Make clear which confounders were adjusted for and why they were included | Not applicable |  |
|  |  | (*b*) Report category boundaries when continuous variables were categorized | Not applicable |  |
|  |  | (*c*) If relevant, consider translating estimates of relative risk into absolute risk for a meaningful time period | Not applicable |  |

| Other analyses | 17 | Report other analyses done—eg analyses of subgroups and interactions, and sensitivity analyses | Table 2 and 3 |  |
| --- | --- | --- | --- | --- |
| Discussion | | | | |
| Key results | 18 | Summarise key results with reference to study objectives | Page 16, lines 400-411 |  |
| Limitations | 19 | Discuss limitations of the study, taking into account sources of potential bias or imprecision. Discuss both direction and magnitude of any potential bias | Page 17-18, lines 440-453 |  |
| Interpretation | 20 | Give a cautious overall interpretation of results considering objectives, limitations, multiplicity of analyses, results from similar studies, and other relevant evidence | Page 21, lines 525-526 |  |
| Generalisability | 21 | Discuss the generalisability (external validity) of the study results | Page 21, lines 433-443 |  |
| Other information | |  | | |
| Funding | 22 | Give the source of funding and the role of the funders for the present study and, if applicable, for the original study on which the present article is based | Submitted during up-load process |  |

*Give information separately for cases and controls in case-control studies and, if applicable, for exposed and unexposed groups in cohort and cross-sectional studies.

**Note:** An Explanation and Elaboration article discusses each checklist item and gives methodological background and published examples of transparent reporting. The STROBE checklist is best used in conjunction with this article (freely available on the Web sites of PLoS Medicine at http://www.plosmedicine.org/, Annals of Internal Medicine at http://www.annals.org/, and Epidemiology at http://www.epidem.com/). Information on the STROBE Initiative is available at www.strobe-statement.org.
